# Falls and long-term care: a report from the care by design observational cohort study

**DOI:** 10.1186/s12875-018-0741-6

**Published:** 2018-05-24

**Authors:** Emily J. Cameron, Susan K. Bowles, Emily Gard Marshall, Melissa K. Andrew

**Affiliations:** 1 Faculty of Medicine, Dalhousie University, Halifax, NS Canada; 2 Department of Pharmacy, Nova Scotia Health Authority-Central Zone, Halifax, NS Canada; 3 Department of Family Medicine, Dalhousie University, Halifax, NS Canada; 4 Department of Medicine (Geriatrics), Dalhousie University, Halifax, NS Canada

**Keywords:** Falls, Long-term care, Nursing homes, Polypharmacy, Potentially inappropriate medication, Frail elderly, Primary health care

## Abstract

**Background:**

Falls and the resulting complications are common among frail older adults. We aimed to explore risk factors and potential prevention strategies for falls in elderly residents of Long-Term Care Facilities (LTCF).

**Methods:**

This was a cross sectional study design using data from the Care by Design (CBD) study, within Nova Scotia’s Capital District Health Authority. This observational time series cohort study collected data before, during and after the implementation of CBD, a new model of coordinated primary care in LTCF. Here, we analyzed data collected after the implementation of CBD (September 1, 2011- February 28, 2012).

**Results:**

Falls were frequent; 56.2% of our sample of 395 residents fell at least once. In univariate analyses, male gender (*p* = 0.009), dementia (*p* = 0.005), and use of Selective Serotonin Reuptake Inhibitors or Selective Serotonin-Norepinepherine Reuptake Inhibitors (SSRI/SNRI) (*p* = 0.084) showed statistically significant associations with having fallen. Benzodiazepine use appeared to be protective for falls (*p* = 0.058). In a fully adjusted multivariable linear regression model, dementia (β coefficient 0.96, 95% CI: 0.83,1.84; *p* = 0.032), visual impairment (β 0.84, 95% CI: 0.13,1.56; *p* = 0.021), and use of any PIMs (β 0.34, 95% CI: 0.037,0.65; *p* = 0.028) were associated with increased risk of having fallen. Benzodiazepine use remained associated with reduced numbers of falls (*p* = 0.009), and SSRI/SNRI use was associated with increased numbers of falls (*p* = 0.007). Male gender was associated with increased falls in the model which excluded frailty (*p* = 0.022), though gender lost statistical significance once frailty was added to the model (*p* = 0.06).

**Conclusions:**

In our sample of LTCF residents, falls were common. Cognitive impairment, male gender, visual impairment, PIM use and use of SSRI/SNRI medications were associated with increased risk of falls, while benzodiazepine use appeared to be associated with a decreased risk of having fallen. Falls remain an important problem among LTC residents. Screening for falls during patient encounters is recommended, along with further research to identify risk factors and target interventions.

**Electronic supplementary material:**

The online version of this article (10.1186/s12875-018-0741-6) contains supplementary material, which is available to authorized users.

## Background

Falls and the resulting complications among the elderly population are a common problem. Among older adults in Canada, ≥ 65 years old, 30% of those living in the community and 50% of residents living in care facilities will fall each year. The complications of falls may/have been found to lead to pain, functional impairment, disability and death in this population [1–3]. Due to the predicted increase in the proportion of elderly people in the population and known complications of falls in this population, it is important to assess the risk factors associated with falls. In assessing individual risk factors, it is important to screen for previous history of falls. One out of three older people fall each year, but less than half of those who fall actually tell their doctor [4]. Falling once doubles one’s chances of falling again, and it also increases the patient’s fear of falling [4, 5].

Prescribed medications have been shown to be an important contributor to falls. Medications such as benzodiazepines, neuroleptics, sedatives, and anti-hypertensive drugs are some of the medications that are known to be associated with increased falls in elderly people [6]. As individuals age they may encounter more co-morbid conditions and higher numbers of medications than younger individuals (reference needed for increased comorbidity and meds). Hence, medications are one of the most important potentially modifiable risk factors for falls among the elderly. Nevertheless, data on the association between falls risk and number of medications taken by Long Term Care facility (LTCF) residents is limited.

Falls in older adults are not only due to extrinsic risk factors such as medications, but also due to intrinsic factors such as cognitive impairment, frailty, gender and age. Cognitive impairment and dementia are known to be associated with increased risk of falls; little is known about other intrinsic risk factors such as gender [7]. Frailty may also be a relevant predictor of falls; it is likely that as people become more frail their poor mobility may predispose to falls, though after a certain point they may have a lower risk of falls if they cease mobilizing [8]. The purpose of this study was to assess and quantify the risk factors associated with falls among older residents of LTCF in Nova Scotia, Canada.

## Methods

### Study design

This was a cross-sectional study using data from the Care by Design (CBD) study, within the Central Zone of the Nova Scotia Health Authority. CBD is a new model of coordinated primary care in LTCF that was implemented in 2009. Prior to CBD model, residents entering LTCF often kept their prior family physician or were responsible for finding a new one. As a result, residents of a single LTCF unit could have been cared for by several different physicians, leading to challenges in team cohesiveness and continuity of care, and lack of coordinated after hours coverage [9]. Key features of CBD included coordinated family physician coverage with a single family physician caring for all residents of a given LTCF floor or unit, regularly scheduled physician visits to the LTCF half a day per week to see residents who require care, clear 24/7 on call coverage, systematic Comprehensive Geriatric Assessment (CGA), and structured medication reviews. The CBD study was an observational time series cohort study which examined changes in healthcare and outcomes for LTCF residents as the new model of coordinated primary care was introduced. The CBD study thus collected data before and after the implementation of CBD. Data were collected from LTCF and, when a resident had an Emergency Health Services 911 call, from the EHS database and acute care Emergency department/hospital chart. For the purpose of our study, data collected during the post-CBD time period (September 1, 2011- February 28, 2012) were used [9]. We selected this time period as it occurred after the implementation of a Long-Term Care Comprehensive Geriatric Assessment tool (LTC – CGA), which provided rich information (e.g., more complete list of diagnoses and functional status) about residents’ health (Additional file 1: Appendix) [10].

### Sample

The sample comprised 395 LTCF residents. This included all of the residents who had an Emergency Health Services (EHS) 911 ambulance call, as well as a random sample of residents without an ambulance call because the overall CBD study had been designed to investigate the impact of the CBD model of care on Emergency Department transfers [11].

### Measures

The number of falls was determined by chart review during a six-month timeframe. For the purposes of analyses using categorical variables, and to distinguish between non-fallers, infrequent fallers, and frequent fallers, falls were categorized as follows: no falls reported (*N* = 171), 1 fall (*N* = 106), 2-4 falls (*N* = 79), 5+ falls (*N* = 34). In regression analyses, the number of falls was treated as a continuous variable. Cognition was measured using both the Mini Mental State Examination (MMSE) score and the clinical diagnosis of dementia, which were both recorded on the LTC-CGA [12]. The LTC-CGA is completed by family physicians every 6 months and after any significant change in health status [10]. Medications were identified from most up to date medication list in the chart, and the research nurse data abstractors used clinical judgment to determine what medications the resident was currently prescribed. Polypharmacy was measured using drug counts and Potentially Inappropriate Medications (PIMs) were identified using the Beers list [13]. Frailty was measured by a clinical frailty scale adapted for use in LTC. Mildly frail individuals have limited dependence on others for instrumental activities of daily living. Moderately frail individuals are those who need help with both instrumental and non-instrumental activities. Severely frail people are completely dependent on other for the activities of daily living. Very severely frail people are completely dependent and are approaching the end of life. Terminally ill individuals have a life expectancy of less than 6 months, who are not otherwise evidently frail [14, 15]. Other risk factors for falls, such as visual impairment, patient transfer and mobility information was also abstracted from the LTC-CGA and/or chart notes [10, 14].

### Analysis

Descriptive statistics included measures of central tendency means and Standard Deviation (SD) for normally distributed variables and medians with Inter Quartile Rage (IQR) where distributions were skewed. Chi Square tests were used for comparison of categorical variables. Linear regression analyses were adjusted for relevant confounders (those with a priori importance such as age, sex, frailty, drug counts) and those drug classes with associations stronger than *p* < 0.2 in univariate analyses. Statistical analyses were done using Stata 8 and SPSS software packages.

### Ethics

The study was approved by the Capital District Health Authority Research Ethics Committee and individual Research Ethics boards of participating LTCF, where these existed.

## Results

Demographic information of the study participants is shown in Table 1. Residents were older (median age = 85 years) and predominantly female. Of the 285 individuals where sufficient data was collected to estimate frailty, approximately half (52%) were severely or very severely frail.

**Table 1:** Resident characteristics

Resident Characteristics	*N* = 395
Age (years)	Median (IQR)
Median age of residents	85 (77 – 90)
Sex	% (N)
% Female	68.1 (269)
Martial Status	% (N)
Married	16.7 (66)
Single	11.9 (47)
Divorced	8.6 (34)
Widowed	38.5 (152)
Unknown	24.3 (96)
Vision	% (N)
Impaired	39.1 (108)
Frailty	% (N)
Mild	7.6 (30)
Moderate	27.1 (107)
Severe	32.7 (129)
Very Severe	4.1 (16)
Terminally ill	0.8 (3)
Missing data	27.8 (110)
Cognition	%(N)
Dementia	64.1 (253)

**Fig. 1:**
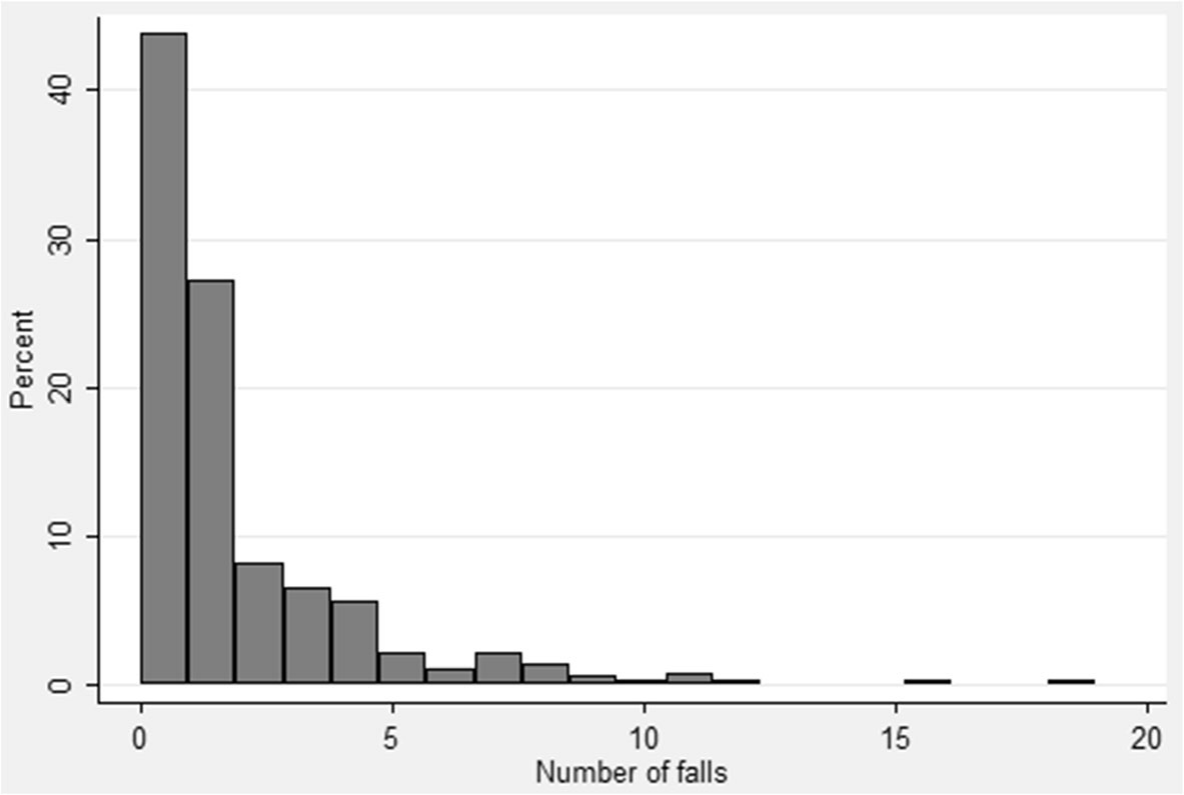
Total number of Falls Reported per Resident

Of the 395 residents included in the analysis, 390 residents had falls data. The distribution of falls is shown in Fig. 1.

### Univariate analyses

Table 2 shows the results of univariate analysis. Male gender was a significant predictor of falls (*p* = 0.009). Further analysis of gender and falls category showed a greater percentage of men fell more often. Among the men in the study, 14.5% were reported to have fallen five or more times compared to 6.1% of women (*p* = 0.04).

**Table 2:** Associations of individual variables with falls (univariate analyses)

Factor	β value (95% CI)	*P* value
Male Gender	−0.69 (−1.20, −0.17)	0.009
Dementia	0.85 (0.26, −1.43)	0.005
Increased Frailty	−0.16 (− 0.54- 0.21)	0.39
Benzodiazepines	−0.47 (− 0.96, 0.17)	0.058
SSRI	0.43 (−0.58, 0.91)	0.084

There were more overall falls in residents with dementia than those without (63.35% vs. 41.84%); this difference was statistically significant (β=0.85**,***p* = 0.005). For example, a group of 10 residents with dementia would experience 8 falls more than a group of 10 residents without dementia. Looking at the highest rate of falls, 10.8% of residents with dementia fell 5 or more times, compared with 6.1% of residents without dementia (*p* = 0.001).

The number of residents taking each individual class of medication as well as the relationship between fall categories and medication by class is shown in Table 3. There was a trend towards residents taking benzodiazepines having fewer falls compared to those not taking benzodiazepines (*p* = 0.058), and use of SSRIs was associated with increased risk of falls (*p* = 0.084).

**Table 3:** Medication class in relation to number of falls

Medication	N of residents taking each med. *N* = 395	0 (*N* = 171)	1 (*N* = 106)	2-4 (*N* = 79)	5+ (*N* = 34)	*P*-value
Typical antipsychotics	30	9 (30%)	9 (30%)	8 (27%)	4 (13.3%)	0.4
Atypical antipsychotics	156	66 (42.3%)	41 (26.3%)	33 (21.2%)	14 (9.0%)	0.96
SSRI/SNRI	219	97 (44.3%)	47 (21.5%)	51 (23.3%)	22 (10.0%)	0.03
Diuretic	193	85 (44.0%)	52 (26.9%)	36 (18.7%)	16 (8.3%)	0.94
Beta blocker	186	82 (44.1%)	48 (25.8%)	36 (19.4%)	17 (9.1%)	0.95
CCB	104	41 (39.4%)	29 (27.9%)	23 (22.2%)	8 (7.7%)	0.81
ARB	54	27 (50%)	11 (20.3%)	12 (20.45)	2 (3.7%)	0.31
Opioid	211	92 (43.65)	61 (28.9%)	41 (19.4%)	17 (8.1%)	0.83
Statin	149	68 (45.6%)	39 (26.2%)	30 (20.1%)	10 (6.7%)	0.72
Diabetic	106	45 (42.5%)	33 (31.1%)	20 (18.95)	7 (6.6%)	0.62
Bisphosphonate	64	28 (43.8%)	13 (20.3%)	17 (26.6%)	6 (9.4%)	0.41
Benzodiazepines	164	74 (45.1%)	53 (32.3%)	26 (15.9%)	10 (6.1%)	0.05

*CCB* Calcium Chanel Blocker, *ARB* Angiotensin Receptor Blocker, *SSRI/SNRI* Selective Serotonin Reuptake Inhibitors or Selective Serotonin-Norepinepherine Reuptake Inhibitors

Further analysis was done to explore why benzodiazepine appeared to be protective for falls, particularly in relation to End of Life and mobility status. 54% of residents who were classified as end of life were on benzodiazepines, which equated to 26.8% of all benzodiazepine users being at end of life (*p* = 0.009). 47.5% of those taking benzodiazepines had independent transfers, while 32.8% were completely dependent for transfers (*p* = 0.036). 57.9% of residents on benzodiazepines were walking independently, while 34.2% were completely dependent with walking (*p* = 0.032).

In univariate analysis, frailty was not significantly associated with falls (*p* = 0.39). Table 4 shows associations between falls and frailty, classified using the frailty scale. There were no significant associations between frailty and gender (*p* = 0.21), frailty and falls categories (*p* = 0.95), or frailty and benzodiazepine use (*p* = 0.11).

**Table 4:** Frailty in relation to gender, benzodiazepine use and number of falls

	1- mild frailty	2 - moderate frailty	3 - severe frailty	4 – very severe frailty	5 – terminally ill	*P* value
Male Gender *N* = 92	11 (12%)	40 (43.4%)	33 (35.9%)	6 (6.5%)	2 (2.2%)	0.21
Female Gender *N* = 192	19 (10%)	67 (34.9%)	95 (49.5%)	10 (5.2%)	1 (0.5%)	0.21
Benzodiazepine Use *N* = 118	13 (11.0%)	42 (35.6%)	59 (50%)	2 (1.7%)	2 (1.7%)	0.11
0 Falls *N* = 113	14 (12.4%)	41 (36.3%)	50 (44.3%)	7 (6.2%)	1 (0.9%)	0.95
1 Fall *N* = 74	6 (8.1%)	27 (36.5%)	36 (48.6%)	4 (5.4%)	1 (1.4%)	0.95
2-4 Falls *N* = 70	5 (7.1%)	30 (42.9%)	31 (44.3%)	3 (4.3%)	1 (1.4%)	0.95
5+ Falls *N* = 27	5 (18.5%)	9 (33.3%)	11 (40.7%)	2 (7.4%)	0	0.95

### Multivariate analyses

**Fig. 2:**
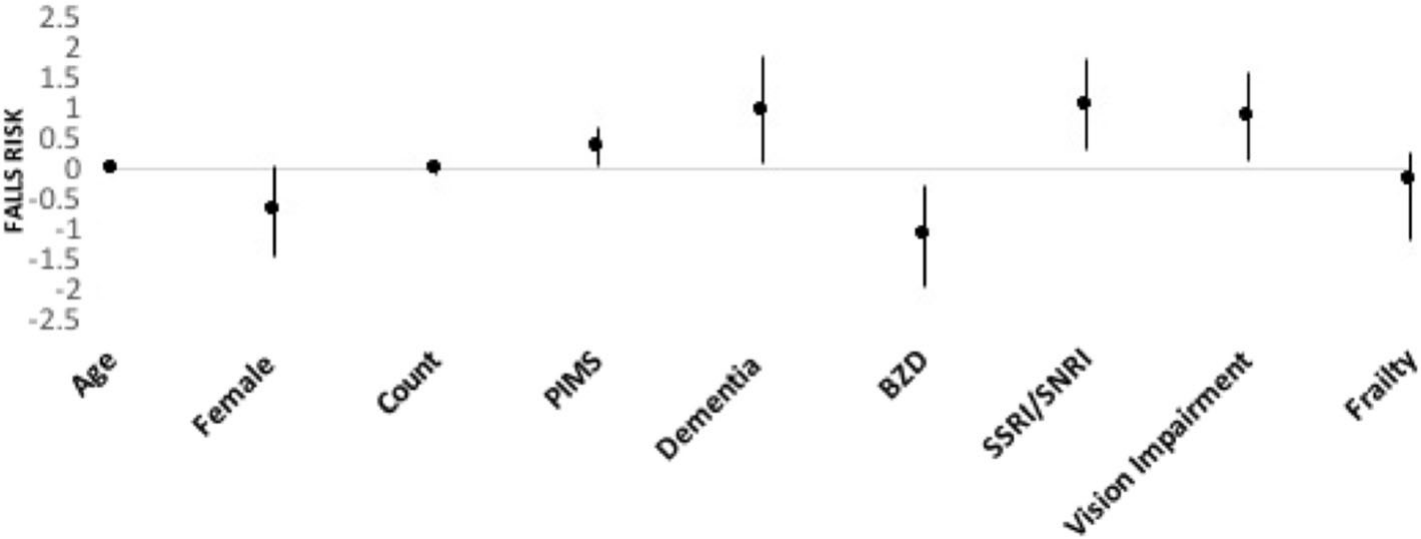
Individual variables in relation to falls risk. Shown are linear regression coefficients with 95% Confidence Interval from the fully adjusted multivariable regression model

A linear regression model was performed, with the dependent variable being the number of falls adjusting for age, sex, dementia diagnosis, drug count, Potentially Inappropriate Medication (PIM) use, visual impairment, frailty, benzodiazepine and SSRI/SNRI use. As shown in Fig. 2, dementia (**β** 0.96, 95% CI: 0.83 – 1.84; *P* = 0.032), visual impairment (**β** 0.84, 95% CI: 0.13 – 1.56; *p* = 0.021), and use of any PIMs (**β** 0.34, 95% CI: 0.037-0.65; *P* = 0.028) were associated with increased risk of falls (Fig. 2). Consistent with our findings in the univariate analyses, benzodiazepine use remained associated with reduced numbers of falls (*p* = 0.009), and SSRI/SNRI use was associated with increased numbers of falls (*p* = 0.007). Age, drug count, and frailty were not significant predictors in this model. Gender was significantly associated with falls in the model which excluded frailty (*p* = 0.022), though it shifted from borderline statistical significance once frailty was added to the model (*p* = 0.06).

## Discussion

Falls were frequent among the frail LTCF residents in our study, with 56.7% of residents having fallen at least once in the prior 6 months. We found that falls were associated with cognitive impairment, male gender, visual impairment and the use of certain medications. Potential Inappropriate Medication use (according to the Beers list) and SSRI/SNRI classes were associated with increased risk of falls, while benzodiazepines appeared to be associated with a decreased risk of falls.

Our results should be interpreted with caution. This was an observational study, and sampling of LTCF residents was not random; those with emergency ambulance calls were over-sampled. Data were abstracted from LTCF charts, and thus we relied on documentation in the charts and on clinical reporting of measures like falls and dementia diagnoses. On the other hand, these results reflect the “real world” of clinical practice. Our study analyzed frailty and falls which is a relationship that has not previously been examined in Long Term Care populations to our knowledge.

Due to the retrospective nature of the study, despite the best efforts of the chart abstractors to glean the relevant data points from resident charts, some residents were missing data. For example, 110 individuals (27.8% of the sample) were missing frailty data because this had not been completed by their primary care teams. Based on prior experience in frailty studies, generally speaking frailty is more likely to be missing in the most vulnerable (who are also the most frail) [16]. Our findings may therefore be conservative, and this may also have contributed to our lack of finding of an association between frailty and falls.

Documentation and reporting of falls is a challenge. Our study did not use a standardized method of recording of falls, as the LTCF did not have a formal tool for recording falls. Falls were counted if they were documented in a resident’s chart upon review of the preceding 6 months. It is possible that fall events would have been missed if they went undocumented (for example if the resident had not reported a fall to the care staff). Potential gaps in reporting of falls are not a problem unique to our study. Reporting differences by gender have been documented in the literature [17, 18]. This is potentially relevant to our study’s finding of an association between male gender and increased fall numbers. The current literature supports that women fall more than men, but this may be due, at least in part, to gender differences in reporting. Stevens et al., examined gender differences in seeking care in the community-dwelling adults over the age of 65. They found that a significantly greater percentage of women than men sought medical care after a falls, talked with a healthcare provider about falls, talked with a provider to understand why they fell, and/or talked with a provider about fall prevention [17]. Since our study was conducted in LTCF, underreporting remains a possibility, though residents would presumably be more likely to come to staff attention when they fell. For this reason, our finding that men in our study had more documented falls appears all the more relevant. Taking our study findings in the context of the existing literature, more focused screening for falls among men may be warranted as a prevention strategy.

The relevance of medication use in relation to falls was another important finding in our study. Our study lacks information on medication doses and indications, which would be beneficial to the interpretation of our findings. In LTCF settings, mediations such as benzodiazepines, antidepressants and antipsychotics have been previously reported to be associated with increased risk of falls [6]. Our finding that SSRI/SNRI and PIMs were associated with increased risk of falls is thus in keeping with the current literature. However, our study found that benzodiazepines appeared to be protective for falls. We conducted further analysis to try and account for this surprising finding. We hypothesized that one potential explanation could be that benzodiazepines are more likely to be prescribed at end of life (when mobility might be restricted and falls risk thus attenuated). However, we found that the majority of benzodiazepine users were not classified as being at end-of life. Another explanation could be that those taking benzodiazepines were less mobile, and therefore less likely to fall, yet we found that over half (57.9%) of the residents on benzodiazepines were walking independently. A third explanation might be the increased education in appropriate prescribing of benzodiazepines among physicians. If residents at high risk of falls preferentially had benzodiazepines de-prescribed or not initiated, while those at lower risk of falls were left taking them, this could have led to an apparently inverse association. This is particularly interesting in our study as during the time period prior to our data collection, the CBD team implemented new prescribing guidelines and medication review protocols for the physicians caring for these patients. The focus was not specifically on benzodiazepines, but it still may have impacted the prescribing habits of the physicians.

Although not statistically significant, the frailty trends discovered in our study are interesting. We found that moderately and severely frail residents experienced more falls. These intermediate frailty categories were also were the highest benzodiazepine users. Moderate to severe frailty could be considered the most vulnerable group to experience falls, as they could be mobile but also dependent for most basic activities of daily living [14]. The extremes of frailty are likely to cancel each other out, with opposite influences. Very frail and terminally ill residents might tend to be bed bound (and thus at lower risk of falls) while those who with mild frailty would not be as likely to fall as they presumably have greater independence with mobility.

Generally speaking, women tend to be more frail than men [15]. Our lack of finding of an association between gender and frailty may reflect a placement bias. Less frail men may be more likely to be cared for at home by their surviving wives, moving to LTC only at with more advanced frailty. Women may have to move to LTC at lower levels of frailty if they have been widowed and have fewer options for care at home [19].

Our study also found visual impairment and impaired cognition to be correlated with increased risk of falls. The current literature lacks studies associating visual impairment and falls in residents of LTCF. Unfortunately, our study can only conclude visual impairment increased the risk of falls, but cannot comment of the specific degree or type of visual impairment, because we relied on a general clinical diagnosis of “visual impairment”. However, visual impairment is something that is potentially modifiable, so it should not be overlooked as a risk factor for falls. On the other hand, dementia is a well-documented risk factor for falls [20]. Our study findings support the importance of dementia as a risk factor for falls in LTCF. Frequent cognitive screening and staging, and falls reduction interventions targeted to residents with dementia, may help to implement preventative measures.

## Conclusions

Our study examined modifiable and non-modifiable risk factors for falls among LTCF residents. Keeping in mind the study limitations, we found that certain medications, impaired cognition, and visual impairment were associated with increased falls risk. This is consistent with the current literature and supports the need for screening and prevention of these risk factors. Screening for falls and associated risk factors should ideally occur during every patient encounter. Our finding that men fell more than women is a unique finding that requires more definitive research. Based on our findings, clinicians should consider asking men explicitly about falls as they may be less likely to be forthcoming. The finding that benzodiazepines were protective for falls may in fact reflect appropriate changing prescribing patterns of clinicians (e.g. benzodiazepines being stopped in those at risk for falls and started at end of life). Falls remain an important problem among LTC residents and further research is needed to inform clinical practice.

## Additional file

Additional file 1: Appendix A. Comprehensive Geriatric Assessment tool. This is the Appendix figure showing the LTC-CGA tool which was used as part of the Care by Design model. (PDF 55 kb)

## Abbreviations

ARB: Angiotensin Receptor Blocker
CBD: Care by Design
CCB: Calcium Chanel Blocker
CGA: Comprehensive Geriatric Assessment
CI: Confidence Interval
EHS: Emergency Health Services
IQR: Inter Quartile Rage
LTC: Long-Term Care
LTCF: Long-Term Care Facilities
MMSE: Mini Mental State Examination
PIM: Potentially Inappropriate Medication
SD: Standard Deviation
SSRI/SNRI: Selective Serotonin Reuptake Inhibitors or Selective Serotonin-Norepinepherine Reuptake Inhibitors
