# Supplementary material for: Falls and long-term care: a report from the care by design observational cohort study
Source: BMC Fam Pract. 2018 May 24;19:73. doi: 10.1186/s12875-018-0741-6 (PMC5968500; doi:10.1186/s12875-018-0741-6)
Supplement: Supplementary file 1 — Appendix A. Comprehensive Geriatric Assessment tool. This is the Appendix figure showing the LTC-CGA tool which was used as part of the Care by Design model. (PDF 55 kb) [file 12875_2018_741_MOESM1_ESM.pdf]

Capital Health

Appendix A

Long-Term Care Comprehensive Geriatric Assessment Form

WNL = Within Normal Limits   ASST = Assisted
IND = Independent   DEP = Dependent

Cr Cl/eGFR: ________________

Chief lifelong occupation:_______________   Education: (years)______

Cognitive Status
☐ WNL
☐ Dementia
☐ Delirium
MMSE ________
Date___________

Emotional
☐ WNL ☐ ↓Mood
☐ Depression ☐ Anxiety
☐ Other
☐ Hall/Delusions

Behaviours
☐ Verbal Non-aggressive
☐ Verbal Aggressive
☐ Physical Non-aggressive
☐ Physical Aggressive

Infection Control
☐ MRSA
☐ VRE
☐ Flu shot given
☐ Pneumococcal vaccine given
☐ TB test done
☐ Tetanus/Diphteria

Communication:

Speech	Hearing	Vision
☐ WNL	☐ WNL	☐ WNL
☐ Impaired	☐ Impaired	☐ Impaired

Footcare needed ☐ Y ☐ N
Dental care needed ☐ Y ☐ N
Skin Integrity Issues ☐ Y ☐ N

Strength
☐ WNL ☐ Weak Upper: PROXIMAL DISTAL R L
Lower: PROXIMAL DISTAL R L

**Mobility**	Transfers	IND ASST Dep
	Walking	IND Slow ASST Dep
	Aid	____________________
**Balance**	Balance	WNL Impaired
	Falls	N Y Frequency
**Elimination**	Bowel	CONSTIP CONT INCONT
	Bladder	CATHETER CONT INCONT
**Nutrition**	Weight	STABLE LOSS GAIN
	Appetite	WNL FAIR POOR
**ADLs**	Feeding	IND ASST Dep
	Bathing	IND ASST Dep
	Dressing	IND ASST Dep
	Toileting	IND ASST Dep

Legal NoK:________________________

Advanced Directives ☐ Y ☐ N

Code Status
☐ Do Not Attempt to Resuscitate
☐ Do Not Hospitalize
☐ Resuscitate

Marital Status
☐ Married
☐ Divorced
☐ Widowed
☐ Single

Family Stress
☐ None
☐ Low
☐ Moderate
☐ High

Problems/Past history	Medication adjustment required. Associated Medication
1. ______________	❍ ______________
2. ______________	❍ ______________
3. ______________	❍ ______________
4. ______________	❍ ______________
5. ______________	❍ ______________
6. ______________	❍ ______________
7. ______________	❍ ______________
8. ______________	❍ ______________
9. ______________	❍ ______________
10. ______________	❍ ______________
11. ______________	❍ ______________
12. ______________	❍ ______________
13. ______________	❍ ______________
14. ______________	❍ ______________
15. ______________	❍ ______________

Current Frailty Score
Scale
1. Mildly frail ☐ 2. Moderately frail ☐ 3. Severely frail ☐ 4. Very severely ill ☐ 5. Terminally ill ☐

Physician: ______________________________ Date: ____________
YYYYMMDD

CD0184MR_06_

© 2007-2008 All rights reserved. Geriatric Medicine Research, Dalhousie University.Halifax, Canada.
Used with permission
